# Cancer—Practical Conclusions

**Published:** 1875-04

**Authors:** 


Cancer—Practical Conclusions.—Carcinoma and sarcoma are probably both local diseases in the outset, and could they be excised or otherwise destroyed sufficiently early, would result in a permanent cure. The fact is, however, that in carcinoma germs have usually been sent along the lymphatics, beyond the reach of the surgeon, long before the patient presents himself for treatment; hence, the removal of the original tumor does not prevent the recurrence in the lymphatic glands. The cancer, however, ought to be removed, and the earlier the better; and it would seem to be our duty at the same time to remove the corresponding lymphatic glands, even if they are not perceptibly enlarged, because their infection may be considered as already accomplished, though the development be not yet manifest. There is no rational doubt that early and thorough extirpation of the cancer on the average prolongs life and comfort.

The removal of sarcoma is a much more hopeful procedure. The glands not being infected, the patient often show little tendency to a recurrence after a thorough excision, so that an early and complete removal is above all things to be desired.—*Medical Examiner.*

